# Clinical and Epidemiological Profile and Treatment Outcomes of Tuberculous Cervical Lymphadenitis: A Hospital-Based Observational Study

**DOI:** 10.7759/cureus.99524

**Published:** 2025-12-18

**Authors:** Praveen Nayak, Vishnukanth Govindaraj, Dharm Prakash Dwivedi, Noyal M Joseph, T. P. Elamurugan, Madhusmita Mohanty Mohapatra, Manju Rajaram, Pratap Upadhya, Vinod Kumar Saka

**Affiliations:** 1 Pulmonary Medicine, Jawaharlal Institute of Postgraduate Medical Education and Research (JIPMER), Puducherry, IND; 2 Medical Microbiology, Jawaharlal Institute of Postgraduate Medical Education and Research (JIPMER), Puducherry, IND; 3 Surgery, Jawaharlal Institute of Postgraduate Medical Education and Research (JIPMER), Puducherry, IND; 4 Internal Medicine, Jawaharlal Institute of Postgraduate Medical Education and Research (JIPMER), Puducherry, IND

**Keywords:** cbnaat, extra-pulmonary tuberculosis (eptb), non-tuberculosis mycobacteria, paradoxical reaction, tb lymphadenits

## Abstract

Background: Tuberculosis (TB) continues to be the biggest health problem in developing countries with enormous social and economic implications. The commonest form of extrapulmonary TB (EPTB) is tubercular cervical lymphadenitis, or scrofula. The diagnosis and management of TB lymphadenitis is challenging, given the multiple differentials. Also, relapse and persistence of lymph nodes (LNs) despite treatment result in significant morbidity.

Methodology: A hospital-based descriptive study was conducted among 35 newly diagnosed cervical tubercular lymphadenitis patients who were initiated on anti-tuberculous treatment (ATT). Patients were followed up till treatment completion and six months post-treatment to rule out relapse.

Results: The most common clinical presentation was painless neck swelling. The most common comorbidities noted were hypertension (*n* = 2) and hypothyroidism (*n *= 2). Chronic kidney disease and diabetes mellitus were observed in one patient each. Two patients also had pulmonary TB. Six months of ATT were advised for all the patients initially. Thirty-four patients completed treatment. One died during the fourth month of treatment. Eighteen patients had complete resolution of LNs, and three patients required extension of treatment. No paradoxical reactions or drug-induced hepatitis were observed.

Conclusions: Extrapulmonary TB (EPTB) contributes to one-third of cases of TB. Microbiological confirmation of TB is essential even when pathological confirmation exists. Patients with cervical adenitis show an excellent response to first-line ATT, and six months of treatment is sufficient. Post-treatment follow-up for six months revealed no relapse. The study results need to be confirmed in a larger sample size.

## Introduction

Globally, tuberculosis (TB) is a major public health issue causing significant morbidity and mortality. This is more evident in the emerging nations. As per the World Health Organization (WHO) global report 2020 [1], every year globally close to 9.6 million individuals get infected and 1.5 million die from TB. During the COVID-19 pandemic, there were significant disruptions in TB services, resulting in a decline in TB incidence. The reported number of people newly diagnosed with TB fell from 7.1 million in 2019 to 5.8 million in 2020 and 6.4 million in 2021. Post-pandemic, there is a significant rise in TB incidence, with the WHO global TB report revealing an estimated global incidence of 10.8 million in 2023 [2]. The post-pandemic rise in TB incidence reflects the consequence of disruptions to TB diagnosis and treatment during the COVID-19 pandemic.

The eight countries ranked in order from first to last in terms of numbers of cases, and that accounted for about two-thirds of global cases in 2022, are India, Indonesia, China, the Philippines, Pakistan, Nigeria, Bangladesh, and the Democratic Republic of the Congo [2]. However, the incidence of TB in India has shown a decreasing trend. From 237 per lakh population in 2015, it has decreased to 199 per lakh population in 2022, with a 16% decline [3]. The TB India Report 2024 observed that nearly 20%-24% of all TB cases are extrapulmonary (EPTB), with tuberculous lymphadenopathy being the most common type, accounting for approximately 35% of all EPTB cases [4].

Even with the advent of newer diagnostic methods, the diagnosis of TB lymphadenitis is a challenging one. The commonly employed tests for TB lymphadenitis include lymph node (LN) aspirate for nucleic acid amplification testing (NAAT) and histopathological demonstration of granulomas. Cartridge-based NAAT (CBNAAT) can have a variable diagnostic yield of approximately 50%-80% [5,6], and the demonstration of granulomas on histopathology is not specific for TB, as it may also be seen in conditions such as non-tuberculous mycobacterial disease, sarcoidosis, toxoplasmosis, tularemia, fungal infections, and cat-scratch disease [7].

TB lymphadenitis poses a few challenging conditions during treatment, like the formation of new LNs, an increase in the size of already existing LNs, the development of fluctuation, sinus tracts, and the persistence of LNs after treatment completion. Similar to pulmonary TB (PTB), drug resistance also poses a problem in the management of EPTB. A meta-analysis observed that adenitis is the commonest form of drug-resistant EPTB, with studies reporting 21% to 47%. The prevalence of multidrug-resistant (MDR) EPTB was approximately 3%-4% [8]. With the above points in mind, we undertook this research to study the clinical profile and epidemiological factors of tubercular cervical lymphadenitis, and to determine and assess the outcome of anti-tuberculous treatment (ATT) in these patients.

## Materials and methods

This descriptive study was conducted at a tertiary care institute in southern India from July 2020 to December 2021 after obtaining scientific and ethics approval.

Persons diagnosed with cervical lymphadenitis of TB etiology were included in the study. TB lymphadenitis was diagnosed by one or more of the following methods: histopathology, cytology, CBNAAT, or acid-fast bacilli (AFB) staining. In the absence of a microbiological or tissue diagnosis, patients who were started on ATT based on strong clinical suspicion and at the clinician’s discretion were also included, in accordance with recommended guidelines. Patients already receiving anti-tubercular treatment were excluded.

The sample size was calculated by assuming that the proportion of individuals with tuberculous cervical lymphadenitis who develop paradoxical reactions after starting ATT is 20%, as reported by Seok et al. [9]. With an alpha error of 5% and 20% relative precision, the required sample size was estimated to be 160. A review of our hospital records for the two years preceding the study showed that approximately 160 patients with TB lymphadenitis seek treatment per year. Anticipating a paradoxical reaction rate of 20%, we calculated 20% of 160 cases for sampling purposes, arriving at a final sample size of 35. 

Sampling technique: Consecutive sampling

Consecutive patients diagnosed with TB cervical adenitis and referred to the DOTS center in the Department of Pulmonary Medicine for initiation of anti-tubercular treatment were enrolled in the study.

Prestructured proforma

The details, including clinical features, epidemiological information, and the methods of diagnostic tests, were entered into the proforma (Appendix).

Data collection methodology

Consecutive patients with tuberculous lymphadenitis who met the inclusion criteria were enrolled in the study after providing informed consent. Their sociodemographic details were collected and noted in a proforma. History was taken from patients and attenders. Important history regarding the site of disease and diagnostic methodologies, including fine needle aspiration cytology (FNAC), excision biopsy, fluorescent microscopy of LN aspirate, CBNAAT, Mantoux test, and chest X-ray, was noted. Before the start of ATT, baseline liver function tests, renal function tests, and HIV serology were collected. Comorbid illnesses were recorded.

These patients were started on ATT as per the National Tuberculosis Elimination Program (NTEP) of the Government of India. The proposed treatment was six months of ATT, comprising two months of an intensive phase consisting of isoniazid, pyrazinamide, rifampicin, and ethambutol, followed by four months of a continuation phase with three drugs: isoniazid, rifampicin, and ethambutol. All these drugs were given as fixed-dose combinations daily, based on body weight. All patients were followed up by phone calls every two months until completion of treatment.

After completion of six months of treatment with ATT as per guidelines, patients were assessed physically for the resolution of LNs. If no LN was palpable or if the LN size was decreased by more than 50% of the pretreatment size, ATT was stopped. If there was a persistence of LN or if the size reduction was less than 50% of the pretreatment size, the initial diagnosis was revisited, and the LN was subjected to gene expert and also for identification of non-tubercular mycobacteria by polymerase chain reaction. The maximum duration of extension of ATT was for three months. After completion of treatment, all the patients were followed up every third month for a six-month post-treatment period. During the follow-up period, patients were evaluated for the appearance of new LNs and were asked about the four-symptom complex of TB, namely persistent cough, fever, night sweats, and weight loss.

The collected data were coded, entered into a Microsoft Excel worksheet, and exported to SPSS. Data were analyzed using SPSS version 21 (IBM Corp., Armonk, NY). The results are presented as percentages in categories and displayed in tables and diagrams.

## Results

Demographics of the study population

The majority of the subjects were young, aged 21-30 years. There was also a female preponderance with a male:female ratio of 0.5:1 (Table 1). The patients reported multiple symptoms, and the most common clinical presentation was painless neck swelling (28, 80%). Other associated symptoms included fever, loss of appetite, and loss of weight (Table 2).

**Table 1:** Age and gender distribution of the study population.

Age group	Frequency (*n*)	Percentage (%)
<10 years	1	2.9%
11-20 years	3	8.6%
21-30 years	15	42.9%
31-40 years	6	17.1%
41-50 years	6	17.1%
51-60 years	4	11.4%
Total	35	100%
Age (Mean ± SD) (years)	32.1 ± 12.8	
Gender		
Male	13	37.1
Female	22	62.9
Total	35	100
Sex ratio (M:F)	0.5:1	

**Table 2:** Symptoms noticed in the study population.

Signs and symptoms	Frequency (*n* = 35)	Percentage (%)
Swelling	35	100
Pain	7	20
Fever	3	8.5
Loss of appetite	5	14.2
Loss of weight	7	20
Discharging sinus	0	0
Multiple symptoms (swelling, pain, fever)	3	8.5

LN characteristics

Our patients were examined clinically for the group of LN involvement, consistency, number, pain, and tenderness. The most common involved cervical group of LNs was level 5 - supraclavicular (*n* = 28). Three patients had involvement of multiple groups of cervical LNs.

On assessing the consistency of LNs, the majority of patients (*n* = 26) had single, hard LNs on examination. Tenderness was observed in seven patients.

Comorbid illness

In our study population, patients were also evaluated for the presence of comorbidities. The most common comorbidities noted were hypertension (*n* = 2, 5.7%) and hypothyroidism (*n* = 2, 5.7%). Chronic kidney disease (CKD) and diabetes mellitus were observed in one patient each (2.8%; Table 3). None of our patients had associated Human Immunodeficiency Virus ( HIV) infection. 

**Table 3:** Distribution of comorbid illnesses in the study population. CKD, chronic kidney disease

Comorbidities	Frequency (*n* = 35)	Percentage (%)
Diabetes	1	2.8
Hypertension	2	5.7
Hypothyroid	2	5.7
CKD	1	2.8
HIV	0	0

Investigations

Diagnosis of TB lymphadenitis was based on FNAC of the LN. All patients initially underwent FNAC to establish the diagnosis, as it is a standard practice at the institute. The aspirated material was sent for cytology, AFB smear, and CBNAAT. In 33 of 35 patients (94%), cytology showed caseating granulomatous lymphadenitis. In seven patients (20%), CBNAAT was positive for Mycobacterium tuberculosis (MTB) and sensitive to rifampicin. In two patients, FNAC was inconclusive, and their CBNAAT was also negative for MTB. These two patients underwent excision biopsy, and the sample was sent for histopathology and CBNAAT. Their biopsy showed caseating granulomatous lymphadenitis, and CBNAAT was positive for MTB with rifampicin sensitivity. The results of these investigations are presented in Table 4.

**Table 4:** Investigations performed in the study population. CBNAAT, cartridge-based nucleic acid amplification test; FNAC, fine-needle aspiration cytology; AFB, acid-fast bacilli

Investigations	Frequency, *n*	Percentage (%)
CBNAAT of FNAC		
Positive	7	20%
Negative	28	80%
Total	35	100%
Liquid culture of FNAC material		
Positive	-	-
Negative	35	100%
Total	35	100%
Smear for AFB of FNAC material for microscopy		
Positive	-	-
Negative	35	100%
Total	35	100%
Chest X-ray		
Normal	33	94.3%
Abnormal	2	5.7%
Total	35	100%

All patients with cervical tubercular lymphadenitis were also evaluated for PTB, and two patients were diagnosed with rifampicin-sensitive, sputum smear-positive PTB. 

All the patients were started on ATT as per guidelines. One patient with preexisting CKD was started on an alternate-day dose of ethambutol based on creatinine clearance. After starting ATT, patients were followed up during their scheduled visit, and adverse events were noted.

Adverse events

After starting on ATT, patients were followed up for compliance and adverse events. The most common adverse effect was vomiting, observed in nine patients (25.7%), followed by gastritis in seven patients (20%). The adverse events are depicted in Table 5. These symptoms were observed within the first two weeks of starting ATT. These patients were evaluated for possible ATT-induced hepatitis. Their liver function tests (LFTs) were normal, and they had no clinical signs of jaundice. Therefore, they were continued on ATT with periodic monitoring. Two patients developed a skin rash, one of whom had a severe reaction induced by rifampicin. His ATT was stopped, and rifampicin was replaced with moxifloxacin for the remainder of the treatment course.

**Table 5:** Adverse reactions to ATT. RFT, renal function test (urea, creatinine); ATT, anti-tuberculous treatment

Complaints	Frequency (%)
Vomiting	9/35 (25.7%)
Gastritis	7/35 (20%)
Skin rash	2/35 (5.7%)
Hepatitis	-
RFT derangement	-

Follow-up and outcome of treatment

During the treatment course, patients were followed up periodically by phone at two-month intervals and were asked about the development of new symptoms and their tolerance to ATT. One patient with pre-existing CKD died at the end of the fourth month; the cause of death was attributed to CKD. In our study, no paradoxical reactions were observed. 

At the end of the sixth month after completion of ATT, patients were examined clinically. Eighteen patients showed complete resolution of LNs, in 13 patients the LNs had reduced to less than 0.5 cm, and in three patients the LNs did not regress by more than 50% of their original size. All three patients underwent repeat FNAC and biopsy. Specimens were also sent for nontuberculous mycobacteria (NTM), liquid culture, CBNAAT, and histopathological examination. No NTM infection was noted. Liquid culture was negative for M. tuberculosis, and in all three patients, CBNAAT showed M. tuberculosis with no rifampicin resistance. All three patients had also tested CBNAAT positive before starting ATT. Their treatment was extended by three additional months, and they achieved complete resolution. According to TB treatment guidelines, patients who complete treatment need to be followed up for a period of two years. In the present study, patients were followed every three months for six months post-treatment. During these visits, patients were examined for recurrence of LNs and for any new findings suggestive of TB. None of the patients experienced relapse or recurrence during the six-month follow-up period.

## Discussion

TB lymphadenitis commonly affects the younger age group. The mean age of the study population in our study was 32.1 ± 12.8 years. Studies in high-burden countries such as India by Shetty et al. [10] and in Bangladesh by Kamal et al. [11] also reported predominant involvement in young people. Similar observations have been made in low-burden countries, including by Algarni et al. in Saudi Arabia and by Mathiasen et al. in Denmark [12,13].

In our study, there was a female preponderance. This finding has also been reported in studies by Shetty et al. [10] and Manju et al. [14]. In contrast, a study by Jayakumar et al. [15] in India noted a male predominance. In low-burden countries, the incidence is similar in males and females [13].

The reported incidence of fever in TB lymphadenitis, as noted by Jayakumar et al. and Patel, is approximately 10%-20% [15,16]. We observed associated fever in 8.5% of our patients. Loss of appetite, a common feature of tubercular lymphadenitis, was noted in 14.2% of cases. The incidence of loss of appetite has been reported to be around 5% by Amol et al. [17], but can reach 30%-40% as noted by Algarni et al. [12] and Singh [18]. In patients with cervical adenitis and coexisting HIV infection, the incidence of loss of appetite has been reported to exceed 80% [19]. We observed that only six patients had comorbidities in our study. There were no HIV-positive patients in our study. Studies with larger sample sizes by Seok et al. [9] and Mathiasen et al. [13] reported higher rates of comorbidities. In addition to the smaller sample size in our study, the predominantly younger study population may explain the lower incidence of comorbidities observed.

FNAC is the preferred method over excision biopsy for evaluation of cervical adenitis as it is less invasive and can be performed in our outpatient service. We have noted that FNAC established a diagnosis in more than 90% of cases. The sensitivity and specificity of FNAC in the diagnosis of TB cervical adenitis are reportedly around 80%-90% [5,6]. In the present study, two patients underwent biopsy because FNAC was inconclusive; the diagnosis in both cases was confirmed by biopsy (100%). Biopsy combined with NAAT of the specimen can have a sensitivity of more than 90%, as reported in multiple studies [20,21]. Current NTEP guidelines for the diagnosis of extrapulmonary tuberculosis recommend CBNAAT testing when an adequate specimen can be obtained [22]. CBNAAT testing was performed on the FNA samples of all our subjects. Out of 35 subjects, 7 (20%) tested positive for tuberculosis. This is lower than the reported CBNAAT sensitivity for TB lymphadenitis, which ranges from 60% to 90% [5,6].

There were no treatment defaulters in our study, and better compliance could be attributed to regular telephonic follow-up. Six months of treatment is sufficient for the treatment of TB lymphadenitis, and multiple studies and treatment guidelines also recommend the same [22,23]. Three of our patients required extension of ATT. Studies have reported residual LNs in 20% of cases post-treatment [19]. As noted from our study, after ruling out other possibilities, extension of ATT may suffice in cases with residual LNs.

In our study, the most common side effect of ATT drugs was gastritis (7 patients, 20%), a finding similar to that reported in a few studies [24,25]. One patient developed an allergic skin rash to rifampicin; consequently, rifampicin was replaced with moxifloxacin for six months. A study by Martinez reported that only 0.07% of patients developed skin rashes [26]. However, other studies report that 10-15% of patients experience cutaneous adverse reactions [24,25]. Cases of Stevens-Johnson Syndrome (SJS) and Drug Reaction with Eosinophilia and Systemic Symptoms (DRESS) secondary to ATT have also been documented [27]. None of our patients developed ATT-induced hepatitis. The reported incidence of ATT-induced hepatitis ranges from 8% to 40%. Hepatitis is commonly seen in patients with underlying liver disease [28,29]. Our study population included younger patients with very few comorbidities; this may be one of the reasons for no ATT-induced hepatitis.

A paradoxical reaction is defined as worsening of LN swelling clinically and radiologically, and the formation of new swelling during treatment. Most cases of LN swelling during treatment are due to paradoxical reaction and not relapse, and therefore, do not require retreatment. In our study population, there were no paradoxical reactions noted. The smaller sample size might be the reason for fewer paradoxical reactions noted in our study.

Limitations

Our study had a few limitations. A small sample size of 35 patients may not be extrapolated to a large population. We attempted to follow up with patients by telephone every two months and inquired about any adverse events. As follow-up was conducted every two months, there is a possibility of recall bias for minor adverse events. Additionally, the COVID-19 pandemic affected regular patient visits. Our study population predominantly consisted of younger individuals with relatively few comorbidities. The results may not be extrapolated to individuals with more comorbidities. The smaller sample size may have contributed to the lower number of paradoxical reactions observed in our study.

## Conclusions

TB cervical adenitis is one of the main causes of neck swelling, particularly in young adults. Microbiological confirmation of TB is essential even when pathological confirmation exists. There is an excellent response to first-line ATT in patients with cervical adenitis, and six months of treatment is sufficient. A three-month extension of the continuation phase of ATT may be sufficient in cases of persistent LNs. The possibility of drug resistance or NTM infection should be considered in patients who show no or suboptimal response to ATT, particularly if there was no initial microbiological confirmation. Regular follow-up is essential to ensure treatment compliance. Post-treatment follow-up for six months is necessary to rule out relapse.

## Appendices

Appendix

**Table 6:** Proforma for data collection.

PROFORMA FOR DATA COLLECTION
EPIDEMIOLOGICAL PROFILE:
Name
Age
Sex
Occupation
Address
Marital status
Social status
CLINICAL PROFILE
Presenting symptom and duration
Site of the disease
Clinical examination details
Immunosuppression
HIV status
Diabetes
Co-morbid illness
Weight
BMI
INVESTIGATION PROFILE:
Method of diagnosis
FNAC/CBNAAT/ZN STAINING/HPE/Clinical/PCR
Chest x ray findings
Mantoux test if done
Sputum test if done
TREATMENT DETAILS:
Category of ATT
Adverse events
Drug sensitivity pattern if performed
FOLLOW UP DETAILS:
Second month/fourth month/six month/end of treatment
Follow up procedure: phone call/hospital visit
Increase in existing node size/regression of node size/ appearance of new nodes,/persistence of lymphnode after treatment/residual lymphnode post treatment
Weight gain(quantify)
Improved appetite
BMI post treatment
PERSISTENT LYMPHNODE POST TREATMENT : (EVALUATION ASPECT)
Number of nodes
Size of the nodes
Revaluation needed/ HPE with GENE X PERT/HPE with PCR for NTM
PERSISTENT LYMPHNODE POST TREATMENT : (TREATMENT ASPECT)
Extended treatment
Completely excised
Started on newer regimen
MOTT treatment
